# Exploration of Copper Oxide Nanoneedle Electrosynthesis Applied in the Degradation of Methylene Blue

**DOI:** 10.3390/nano11112994

**Published:** 2021-11-08

**Authors:** Diego P. Oyarzún, Alejandra Tello, Julio Sánchez, Andrés Boulett, Omar E. Linarez Pérez, Rudy Martin-Trasanco, Guadalupe del C. Pizarro, Marcos Flores, César Zúñiga

**Affiliations:** 1 Departamento de Química y Biología, Facultad de Ciencias Naturales, Universidad de Atacama, Copayapu 485, Copiapó 1531772, Chile; alejandra.tello@uda.cl; 2 Departamento de Ciencias del Ambiente, Facultad de Química y Biología, Universidad de Santiago de Chile (USACH), Santiago 9170022, Chile; julio.sanchez@usach.cl (J.S.); andres.boulett@usach.cl (A.B.); 3 Instituto de Investigaciones en Fisicoquímica de Córdoba (INFIQC), Universidad Nacional de Córdoba, Córdoba 5000, Argentina; olinarez@unc.edu.ar; 4 Departamento de Química, Universidad Tecnológica Metropolitana, Avda. Las Palmeras 3360, Santiago 7810000, Chile; ruquim@gmail.com (R.M.-T.); gpizarro@utem.cl (G.d.C.P.); 5 Laboratorio de Superficies y Nanomateriales, Facultad de Física y Ciencias Matemáticas, Universidad de Chile, Beauchef 850, Santiago 8370448, Chile; mflorescarra@ing.uchile.cl; 6 Instituto de Ciencias Naturales, Facultad de Medicina Veterinaria y Agronomía, Universidad de Las Americas, Manuel Montt 948, Santiago 7500975, Chile

**Keywords:** electrosynthesis, CuO/Cu_2_O nanoneedles, photodegradation

## Abstract

In this study, we report a low cost, fast and unexplored electrochemical synthesis strategy of copper oxide nanoneedles films as well as their morphological and chemical characterization. The nanostructured films were prepared using electrochemical anodization in alkaline electrolyte solutions of ethylene glycol, water and fluoride ions. The film morphology shows nanoneedle-shaped structures, with lengths up to 1–2 μm; meanwhile, high-resolution X-ray photoelectron spectroscopy (HRXPS) and spectroscopy Raman analyses indicate that a mixture of Cu(II) and Cu(I) oxides, or only Cu(I) oxide, is obtained as the percentage of water in the electrolyte solution decreases. A preliminary study was also carried out for the photocatalytic degradation of the methylene blue (MB) dye under irradiation with simulated sunlight in the presence of the nanoneedles obtained, presenting a maximum degradation value of 88% of MB and, thus, demonstrating the potential characteristics of the material investigated in the degradation of organic dyes.

## 1. Introduction

The obtention of the nanostructures of different materials has been a subject of increasing interest in the last decade due to their novel physicochemical properties that allow their application in fields such as medicine, acoustics, optoelectronics, photonics, sensors and electrocatalysts [1,2,3,4]. Commonly, nanostructures can be present in various geometries such as wires, rods, points, pores and tubes, among others [4,5].

In addition, nanomaterials produce interesting physical phenomena such as photoluminescence, which make them attractive for the manufacture of electronic devices. The high surface area of these materials could improve the performance of luminescent modules, piezoelectric transducers, semiconductor electrodes, chemical sensors and electrocatalysts [2,5,6]. The most studied nanostructures in the last few years correspond to carbon nanotubes [6], titanium dioxide nanotubes [7] and copper oxides nanostructures [8,9,10]. Among them, copper oxide-based nanomaterials attract attention due to its non-toxic nature, low cost and their low bandgap values of 1.2 to 1.8 eV and 1.8 to 2.5 eV for CuO and Cu_2_O, respectively [11]. Additionally, their conduction and valence bands are close to the water reduction and oxidation potentials, respectively, thus allowing the oxide film to produce hydrogen by direct water photolysis without the need for the application of an external potential. These properties make CuO and Cu_2_O very promising materials for use in converters of solar energy into electrical energy through the design of CuO-based solar cells [12,13] as well as in water photolysis. However, the absorption profile of CuO is rather weak in most of the visible range [14], which implies that if an electrode formed by a smooth CuO film is subjected to solar irradiation, a significant part of the photogenerated charge carriers within the material are lost in recombination processes [15,16]. Nevertheless, as has been demonstrated for other materials such as GaP and SiC [17,18], the use of electrodes composed of nanostructured CuO or Cu_2_O films, could solve this problem. For nanoporous structures, the charge carriers are photogenerated at a small distance from the semiconductor/electrolyte interface, which, in turn, promotes the separation of charge carriers more efficiently [18], while light absorption is reinforced through dispersion [17].

Currently, several methods for obtaining copper oxide-based nanostructures have been widely reported in the literature. In this, the thermal oxidation of metallic copper at high temperatures stands out [19,20], the growth assisted by templates [21,22], the use of colloidal methods [23,24] and using electrochemical anodization techniques. However, high-temperature processes generally limit the control over the interfacial characteristics of copper oxides thin films, which significantly affect their optical and photoelectrochemical properties [25]. Furthermore, the use of templates and routes of colloidal synthesis are generally characterized by obtaining copper oxides that have a low adhesion on the conductive substrates, which limits their use or integration in electronic devices. Conversely, electrochemical anodization is a surface treatment process that has been successfully used to synthesize a wide variety of nanostructures using various metal substrates, such as titanium, zinc, thallium and copper [26,27,28,29,30,31], and employing these films in various applications [32,33,34,35,36]. In particular, the preparation of copper oxide films by anodizing the metallic copper used as a substrate is also the source of the copper ions that are subsequently oxidized by the presence of water molecules composing the electrolyte solution and the voltage applied to the medium [37]. In this context, various morphologies of anodized copper oxide nanostructures, such as nanoneedles, nanorods, nanopores, nanowires and nanoparticles, have been reported [38]. Among these reported morphologies, nanowires stand out due to their high surface area, which increases the reactivity area of the material [13,31]; this offers potential advantages for their integration in electronic devices since they have a much more efficient electrical contact with the metal [13,31], for biomaterial applications or as a non-toxic photocatalyst, low cost and easy to obtain, for the photodegradation of organic molecules, such as dyes and wastewater pollutants [13,31]. Although it has been reported to obtain copper oxide nanoneedles by anodization using an electrolytic medium composed of KOH [31,39,40,41], in no case has an ethylene glycol-based electrolyte medium been used in conjunction with fluoride ions [11,31,39,40,41,42,43]. Tello et al., during 2021, have reported that an electrolytic medium based on the mentioned chemical compounds benefit the morphological order of anodized nanostructures, due to the high viscosity of the solvent and the interference generated by the fluoride ions on the surface of the copper layer during anodization [13,31]. In addition, only one of these studies has used the anodization technique without subsequent heat treatment to dehydrate the Cu(OH)_2_ nanoneedles to CuO [41].

Effluents from the textile, plastic and paper industries contain colorants such as methylene blue (MB) [44], which is a well-known toxic, mutagenic and carcinogenic dye. For this reason, its elimination from wastewater is essential to minimize the effects on aquatic life and the associated problems. In this regard, many conventional methods have been developed for the removal of dyes from wastewater, such as ozonation, liquid–liquid extraction, photodegradation, precipitation, membrane filtration, ion exchange, coagulation and adsorption [45]. However, the advantage of photodegradation compared to these techniques and others used to degrade dyes, such as degradation by algae, enzymes, metal nanoparticles and electrochemistry, is that solar energy is used to produce the reaction that will degrade the dye, which significantly reduces operating costs [46]. To perform the photocatalytic process, a semiconductor material with bandgap energy values Eg < 4 eV is used, which favors the excitation of its valence electrons (VB) toward the conduction band (CB) by means of a light energy source [47].

In this work, we report an unexplored electrochemical strategy synthesis of CuO/Cu_2_O nanoneedles films as well as their chemical and morphological characterization. The anodization was performed in alkaline electrolyte mixtures of ethylene glycol, water and fluoride ions. The film characterization was performed by means of Raman spectroscopy, field emission scanning electron microscopy (FESEM), atomic force microscopy (AFM), transmission electron microscopy (TEM) and HRXPS. Additionally, the previously obtained copper oxide nanostructures were used to perform a study of the photocatalytic degradation of MB in aqueous solution.

## 2. Materials and Methods

Copper anodization was performed in an electrochemical cell with a two-electrode configuration. The anode electrodes consisted of polycrystalline copper foils (Sigma Aldrich (Spruce Street, LA, USA), 99.99% purity) of 250 μm in thickness (0.5 cm^2^ of exposed geometric area) mounted in Teflon holders. A 0.5-centimeter-thick carbon sponge of 3.0 × 3.5 cm^2^ was used as a cathode electrode. Prior to anodization, the samples were mechanically polished using a 0.05-micrometer alumina aqueous suspension and then, degreased by sonication in a 50:50% acetone/ethanol mixture for 15 min. After cleaning, the foils were rinsed with deionized water and dried under N_2_ flux. The anodization was carried out at 5 °C by applying a voltage of 10 using a high-power source, for 180 s. The electrolyte solution was prepared from analytical grade reagents and was based on ethylene glycol + [0.5; 10]% *v*/*v* water, 0.1 M NaOH or 0.1 M KOH and 0.1% *w*/*v* NH_4_F Merck (Darmstadt, Germany).

The morphology features of the nanostructured layers were characterized by FE-SEM using a Carl Zeiss Sigma microscope (Oberkochen, Germany). Raman experiments were performed ex situ (in air) using a Horiba LabRAM HR spectrometer (Sunnyvale, CA, USA), employing a He/Ne laser (632.8 nm wavelength). Using an XPS-Auger Perkin Elmer Model PHI 1257 spectrometer (Waltham, MA, USA) was employed to determine the chemical composition of samples, by means of HRXPS and using the method described previously [48,49,50]. For AFM measurements, a Nanonics Multi View MV1000 was employed using n-type silicon cantilevers (tip radius ≈ 20 nm; f = 39.4 kHz; Q = 1576) for the intermittent mode. TEM was performed using LEO 1420VP equipment (Oberkochen, Germany). Prior to the TEM measurements, a dispersion of copper oxide nanoneedles was obtained by sonication during 15 min of the oxide films in isopropyl alcohol. The analysis of images was performed with the image processing software Gwyddion 2.37. The photocatalytic activity was carried out using a 1000 W Xe/Hg lamp (Oriel 6295) as a simulation of a source of sunlight. In order to avoid the overheating of the MB solution, the infrared radiation was eliminated through a water filter. The copper oxide nanoneedles were immersed in a quartz cell containing 15 mL of a 2.5 mM dye solution. In order to reach the adsorption equilibrium, the samples were held one hour in the dark with permanent air bubbling for also assuring dissolved oxygen saturation at the beginning of the photocatalytic degradation process. The experiments were performed in triplicate for 120 min, and the dye concentration measurements were taken at λ_max_ = 660 nm every 30 min in comparison to a previously recorded calibration curve.

## 3. Results and Discussion

**Figure 1:**
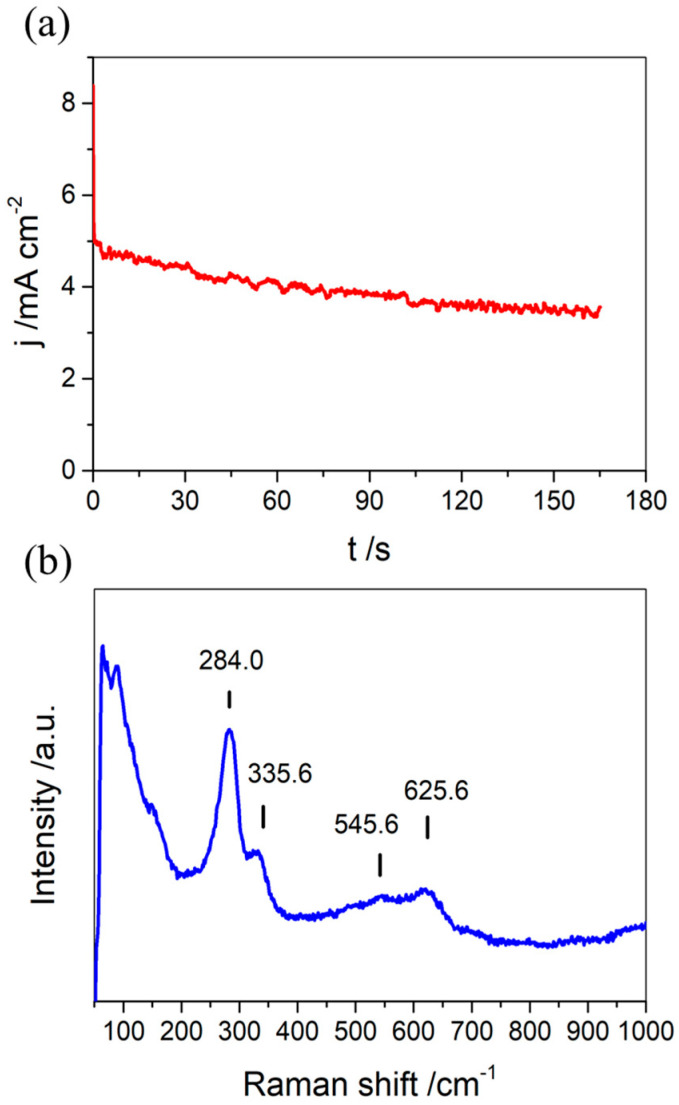
j/t potentiostatic profile (**a**) and Raman spectrum (**b**) for Cu foils anodized at 5 °C applying constant potential of 10 V during 3 min in a 0.1 M KOH + 10%*v*/*v* H_2_O + 0.1%*w*/*v* NH_4_F electrolyte and dissolved in ethylene glycol.

Figure 1a shows the j/t potentiostatic profile applying a constant potential of 10 V during 3 min in a 0.1 M KOH + 10%*v*/*v* H_2_O + 0.1%*w*/*v* NH_4_F dissolved in ethylene glycol solution. After an abrupt initial current density increase, the current profile shows a rapid diminution in current density within the first milliseconds and then a slight variation from around 5 to 3 mA/cm^2^ for the subsequent 3 min due to the passivation of the copper surface [51]. The presence of ethylene glycol and hydroxyl ions in the electrolytic medium may stabilize the current density during the anodization due to its density and polarity as well as the alkaline pH employed [13,31]. On the other hand, the Raman spectrum for the anodized copper foil (see Figure 1b) shows typical intense signals at around 284 and 335 cm^−1^, indicating the presence of CuO species [52,53]. Additionally, signals at 150, 545.6 and 625.6 cm^−1^, which are characteristic of Cu_2_O, are obtained. It should be mentioned that these signals are less intense than those for CuO, which would indicate a low presence of Cu (I) on the surface of the copper foil [52].

**Figure 2:**
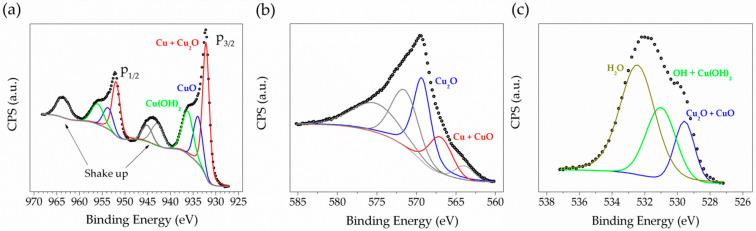
HRXPS spectra of (**a**) Cu-2p, (**b**) Cu-Auger and (**c**) O-1s, for Cu foils anodized at 5 °C applying constant potential of 10 V during 3 min in a 0.1 M KOH + 10%*v*/*v* H_2_O electrolyte + 0.1%*w*/*v* NH_4_F and dissolved in ethylene glycol.

Additionally, the chemical analysis performed using XPS measurements indicates the presence of a Cu_2_O/CuO oxides mixture in the anodized surfaces [37,54]. The high resolution Cu2p spectrum (see Figure 2a) shows important contributions at a binding of 932.2 and 933.9 eV; these signals are attributed to Cu + Cu_2_O, CuO and [49], for the case of the Cu-Auger signal, the Cu + CuO and Cu_2_O contributions are identified at binding energy of 567.0 and 569.3 eV, respectively (see Figure 2b). Additionally, high resolution O1s spectrum (see Figure 2c) evidences the presence of both CuO and Cu_2_O oxides as well as hydrated species in the sample, which are in agreement with the above showed Raman results. Such hydrated species are also identified in the Cu2p main signal at 936.2 eV in binding energy.

Table 1 shows a summary of the results obtained from the XPS measurements; from these values and by using the formulation of Platzman, we estimate the percent for each copper oxidation state [50]. It can be observed that the formation of Cu(I) species predominates in electrode surfaces (46%) after the synthesis process. However, the minor proportion of oxidized Cu(II) species (22%) may be attributed to an excessive amount of H_2_O in the electrolytic bath as well as the high voltage applied during the anodization, given also a high amount of hydrated species, 25%. A small amount of metallic copper (6%) was identified. From the values of this table, the alpha parameter can be determined, which is independent of charge effects, alpha = KE(LMM) + BE(2p) = 917.3 eV + 932.2 eV = 1849.5 eV and 917.3 eV + 933.9 eV = 1851.2 eV. These values are in agree with the literature [55], and in the Wagner plot, can be labeled as Cu_2_O and CuO, respectively.

**Figure 3:**
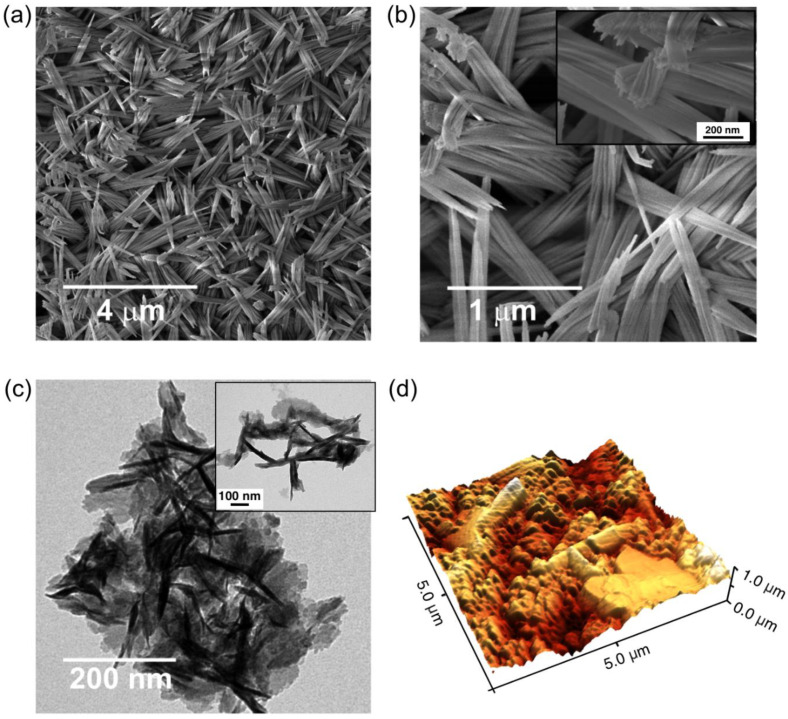
Morphology of anodic CuO/Cu_2_O nanoneedles obtained for Cu foils anodized at 5 °C applying a constant potential of 10 V during 3 min in a 0.1 M KOH + 10%*v*/*v* H_2_O electrolyte + 0.1%*w*/*v* NH_4_F and dissolved in ethylene glycol film obtained by means of (**a**) and (**b**) FESEM, (**c**) TEM and (**d**) AFM.

In order to study the morphology of the obtained films, several microcopy techniques were employed. Figure 3a,b show a top view FESEM micrography of the CuO/Cu_2_O film obtained, which shows a highly homogeneous self-assembled nanoneedles array. The main length of the needles is between ca. 1 and 2 µm. A more detailed study morphology was performed using TEM measurements of a CuO/Cu_2_O nanoneedles dispersion (see Figure 3c). Long and thin structures with lengths ranging between 200 and 500 nm corresponding to the entire and fragmented nanoneedles are observed (see Figure 3c and inset). In addition, Figure 3d shows a 3D AFM image for the anodic films obtained, showing a similar morphology as those obtained from FESEM (see Figure 3a,b).

**Figure 4:**
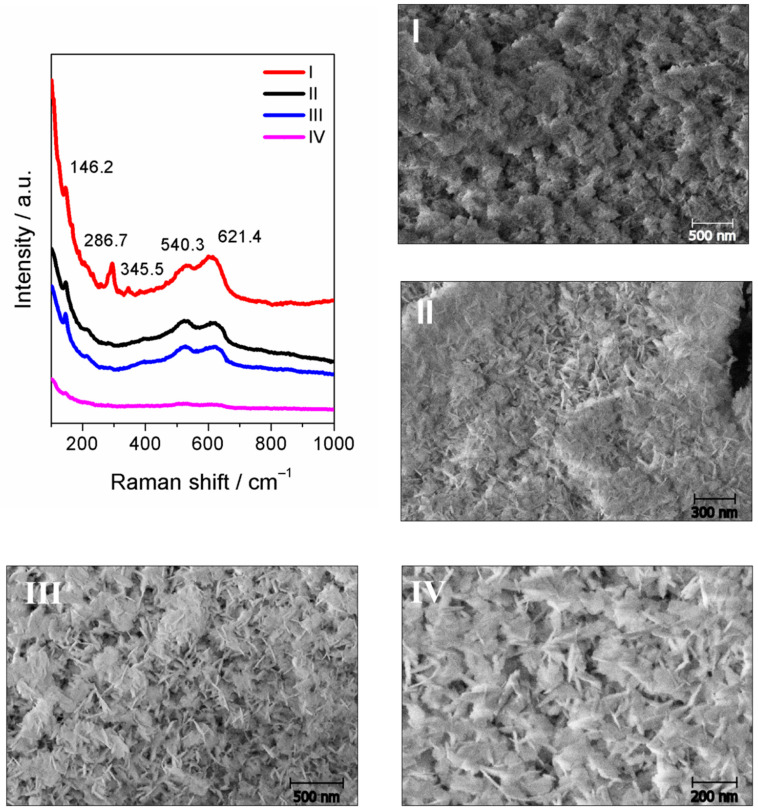
Raman spectra and FESEM images (**I**–**IV**) of copper oxides nanoneedles synthesized by electrochemical anodization at 10 V, for 3 min and 5 °C, using X%*v*/*v* H_2_O (I = 5.0, II = 1.5, III = 1.0, IV = 0.5) + 0.1 M NaOH + 0.1%*w*/*v* NH_4_F, dissolved in ethylene glycol.

**Figure 5:**
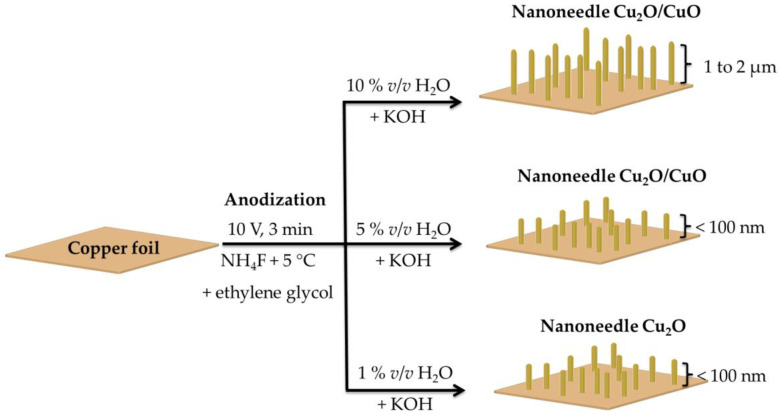
Schematic diagram of the morphology, diameter and chemical composition of the copper oxide nanostructures obtained as a function of the percentage of water used in their respective anodizations.

On the other hand, by decreasing the percentage of water in the electrolyte solution below 10%*v*/*v* during anodization, CuO/Cu_2_O or Cu_2_O nanoneedles are obtained on a flake surface and the nanoneedles have a smaller average length as the percentage of water decreases. The Raman spectra of the nanoneedles obtained using 5%*v*/*v* water in their synthesis show signals at 146.2, 540.3 and 621.4 cm^−1^, which are characteristic of Cu_2_O, together with the signals at 286.7 and 345.5 cm^−1^, which further indicate the presence of CuO in the sample (see Figure 4) [37,52,53,56], whereas, when further reducing the percentage of water in the synthesis, only characteristic signals for the presence of Cu_2_O in the samples are observed [57]. The formation of these nanostructures is explained by the basic medium in which it is found, since the presence of OH^−^, H_2_O and ethylene glycol ions tend to the formation of CuO nanostructures from metallic copper (Equation (1)) [38,40,56]. Moreover, the presence of fluoride ions tends to the redissolution of the CuO previously obtained and the subsequent formation of Cu_2_O (Equations (2) and (3)). Finally, a higher presence of hydroxyl ions when using higher percentages of water again produces CuO from the previously formed Cu_2_O, obtaining a mixture of copper (I) and (II) oxides (Equation (4)) [40,50]. On the other hand, the FESEM images obtained show a highly rough surface made up of a thin film of nanoneedles with diameters apparently below 100 nm. The homogeneity and chemical composition of the synthesized material varies according to the increase in the quantity of water in the electrolytic medium, showing an apparent length diminishing as the water content augments (see Figure 4I–IV). Figure 5 shows a summary diagram of the morphology, diameter and chemical composition of the copper oxide nanostructures obtained as a function of the percentage of water used in their respective anodizations.

Cu_(s)_ + 2OH^−^_(aq)_ → CuO_(s)_ + H_2_O_(l)_ + 2e^−^ (1)

Cu_(sup)_ + F^−^_(aq)_ → Cu-F_(ad)_ + e^−^ (2)

2Cu-F_(ad)_ + 2OH^−^_(aq)_ → Cu_2_O_(s)_ + H_2_O_(l)_+ 2F^−^ (3)

Cu_2_O_(s)_ + 2OH^−^_(__aq)_ → 2CuO_(s)_ + H_2_O_(l)_ +2e^−^ (4)

Based on the discussion of the figures above, it can be deduced that during anodization, a layer of Cu_2_O flakes is generated on the surface of the copper foil, and Cu_2_O or Cu_2_O/CuO nanowires start to emerge on them, depending on the percentage of water used in the synthesis (see Figure 5). This interface, generated between the flakes adhered to the anode surface and the nanowires, allows the nanowires to reach longer lengths as the percentage of water in the synthesis increases [31,37,39,40,41].

**Figure 6:**
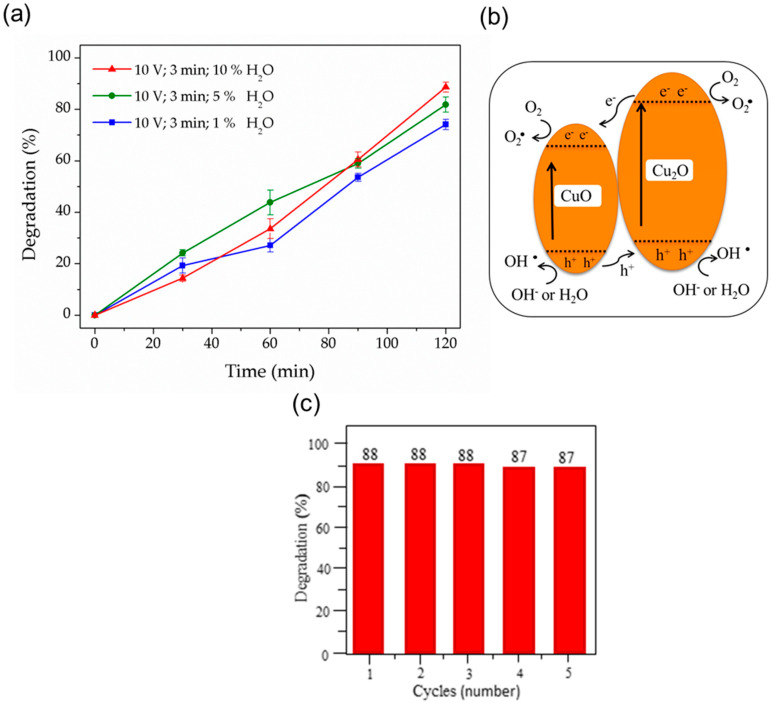
(**a**) Percentage of methylene blue (2.5 mM of MB with 0.001 g/15 mL of CuO/Cu_2_O nanoneedles) degradation as a function of illumination time for different copper oxides nanostructures. (**b**) photodegradation diagram of MB with CuO/Cu_2_O and (**c**) recycling histogram after five cycles of MB degradation.

The photocatalytic activity was evaluated by performing a preliminary study (without adjusting the conditions, such as the amount of xerogel, solution pH, dye concentration and volume) on the degradation of the MB dye, evaluating as a function of the simulated solar irradiation time of CuO/Cu_2_O nanoneedles obtained by anodization with 10 or 3.0%*v*/*v* water in the electrolyte solution during their synthesis, and Cu_2_O nanoneedles obtained by anodization with 1.0% water in the electrolyte solution during their synthesis (see Figure 6a). It is observed that the photocatalytic activity of the CuO/Cu_2_O nanoneedles photocatalysts reaches maximum values of 88 and 82% for the syntheses with 10 and 5% water, respectively; while, for the Cu_2_O nanoneedles obtained with 1% water, in the synthesis it reaches a maximum value of 74%, which indicates that the photocatalytic activity of the materials decreases as a function of the percentage of water used during the synthesis of each photocatalyst, that is, the photocatalytic activity of CuO/Cu_2_O nanoneedles is higher than that of Cu_2_O. This is attributed to the chemical nature of the photocatalyst, since upon irradiation with solar energy (Equation (5)), the electrons (e^−^) and holes (h^+^) generated in the CuO/Cu_2_O heterojunction network have a longer lifetime than those of a Cu_2_O photocatalyst, because the VB and CB of CuO have a lower energy level than the VB and CB of Cu_2_O, respectively, generating a transfer of photoinduced e^−^ from the CB of Cu_2_O to that of CuO and h^+^ from the VB of CuO to that of Cu_2_O and, in turn, increasing the lifetime of the promoted electrons and the photocatalytic activity of the material (see Figure 6b) [11]. Subsequently, these e^−^/h^+^ pairs can react with water molecules in the vicinity of the semiconductor, generating reductive reactions between the promoted electrons and the O_2_ in the water, producing superoxide (O_2_^−^•) (Equations (6) and (7)) and hydroxyl (OH•) radicals (Equation (8)), and oxidative reactions between the holes formed and the OH^−^ coming from the water (Equation (9)) or with the water molecules themselves (Equation (10)), also producing hydroxyl (OH•) radicals [11]. In addition, it has been reported in the scientific literature that the Eg values for Cu_2_O/CuO nanostructures estimated from their UV–Vis absorbance spectra are between 1.5 and 1.9 eV [11], while, for Cu_2_O, it reaches values of between 1.8 and 2.5 eV, i.e., a higher amount of energy is needed to photoinduce e^−^ from VB to CB in a semiconductor composed of Cu_2_O than in a semiconductor composed of a mixture of copper oxides, which also justifies the lower photocatalytic activity obtained for the Cu_2_O nanoneedles [11].

On the other hand, when comparing the photocatalytic activity at lower irradiation times, it is observed that the percentage of MB degradation for the Cu_2_O/CuO nanoneedles obtained with 10% water during the synthesis is lower than those obtained with 5% water during the synthesis, which is attributed to the fact that the photoinduced e^−^ and h^+^ generated in the Cu_2_O/CuO nanoneedles obtained with 10% water have a longer lifetime, but need a longer interaction time between the photocatalyst and the dye to present a significantly higher photocatalytic activity [11].

Semiconductor + hv > Eg → e^−^_(VB)_ + h^+^_(CB)_ (5)

e^−^ + O_2_ → O_2_^−^• (6)

2O_2_^−^• + 2H^+^ → H_2_O_2_ + O_2_ (7)

H_2_O_2_ + e^−^ + H^+^ → H_2_O + OH• (8)

h^+^ + OH^−^ → OH• (9)

h^+^ + H_2_O → H^+^ + OH• (10)

The structural decomposition of MB is mainly caused by the effect of the hydroxyl radicals generated between the reactions of the water molecules with the e^−^ and mainly the h^+^ generated in the photocatalyst. It has been reported that the first thing that is detached from the MB molecules during degradation are the methyl groups attached to the amine group; subsequently, several unstable intermediates are formed with minimum existence periods, until obtaining compounds less complex than the dye, such as SO_4_^2−^, NO_3_^−^, Cl^−^, NH_4_^+^ and mainly H_2_O and CO_2_ [58]. The level of the highest energy occupied molecular orbital (HOMO) and the lowest energy unoccupied molecular orbital (LUMO) of MB have values of 4.25 and 6.11 eV, respectively. MB, when irradiated with an energy higher than 6.11 eV (1.48 × 10^15^ Hz), causes an electron to be promoted from the HOMO to the LUMO, leading to the breaking of a bond and the subsequent degradation of MB [59]. The simulated sunlight used fulfills this characteristic, as it is mainly composed of infrared, visible and, to a lesser extent, UV light (7.5 × 10^14^—3 × 10^15^ Hz), producing a synergistic effect between the degradation of MB due to the UV light portion (approximately 5% of the sunlight) and the free radicals generated in the semiconductor during photodegradation [58]. In repeated photocatalytic degradation tests, the recycling stability of the copper oxide nanoneedles was evaluated for the synthesized system under the experimental conditions of 10 V, 3 min, 10% H_2_O (see Figure 6c). After each repeated cycle, the Cu_2_O/CuO nanostructures were filtered, washed rigorously several times and dried. After five cycles of experiments, the photocatalytic activity of the material continues to show a high degradation index, demonstrating the high stability of this type of copper oxides nanostructures over time, in addition to having all the attributes of reusable materials, to promote sustainable development.

As mentioned above, in this study a maximum photocatalytic activity of 86% was achieved in the degradation of MB using Cu_2_O/CuO nanowires and solar energy. This obtained value is lower than the values previously reported in the scientific literature for various photocatalysts composed of mixtures of metal oxides photo exposed with visible light (see Table 2) [60,61,62,63]. These are attributed to the formation of a heterojunction network between the VB and CB of the metal oxides of different chemical nature, which benefits the lifetime of the photogenerated reactive species [60,61,62,63]. Specifically, Li et al., in 2021, reported the highest photocatalytic activity for MB degradation (99%) using a Cu_2_O/Ag/TiO_2_/polyacrylonitrile nanofiber composite system; however, when studying the material composed only of Cu_2_O nanoparticles stabilized on polyacrylonitrile nanofibers (Cu_2_O/NFs-PAN), a photocatalytic activity of only 60% was reported, which is lower than those obtained in this study [63]. While comparing the materials studied in this research with the standard P25 TiO_2_ degussa applied in the photodegradation of MB, higher or equal photocatalytic activity values were obtained in the preliminary study carried out with anodized Cu_2_O/CuO nanowires, which shows that this material presents potential characteristics to be applied in the photodegradation of organic dyes [43]. On the other hand, the application of CuO nanowires in the photodegradation of various dyes, such as methyl orange (MO), direct red 81 (DR) and victoria blue (VB), have been reported [31,39,40,41,42]. However, these photocatalysts have been obtained by solution chemistry in basic media or by anodization with an electrolytic medium of only KOH [31,39,40,41], and in no case has an electrolytic medium based on ethylene glycol together with fluoride ions been used [11,31,39,40,41,42,43]. The electrolytic medium benefits the morphological order of the anodized nanostructures due to the high viscosity of the solvent and the interference generated by the fluoride ions on the surface of the copper layer during anodization [13,31].

## 4. Conclusions

Based on the results obtained in this research, we conclude that it is possible to synthesize CuO and/or Cu_2_O nanostructures (nanoneedles) using a very simple, one-step and low-cost method. The anodization of copper electrodes in a hydroxyl containing aqueous electrolytic medium, unlike other known methods for the formation of CuO or Cu_2_O nanostructures, does not require special electrolytes, chemicals or surfactants. The chemical characterization of the samples showed that nanowires of Cu_2_O or CuO/Cu_2_O are obtained depending on the conditions of synthesis carried out; in addition, it was determined that nanowires are up to 1–2 μm in length. In the photocatalytic degradation of the methylene blue dye (MB) in the presence of the prepared copper oxide nanostructures, an 88% degradation of MB was obtained for the optimal morphologies. The photocatalytic activity of the material continues to show a high rate of degradation after five cycles of photodegradation, demonstrating the high stability of copper oxide nanostructures over time and the fundamental characteristics of reusable materials. These results are promising for the application of these types of nanostructured copper oxide films for designing new electronic devices with an easy-to-obtain and non-toxic photocatalyst for the photodegradation of organic molecules, such methylene blue dye.

## Figures and Tables

**Table 1:** Cu-2p and O-1s data table of XPS spectra, for Cu foils anodized at 5 °C applying a constant potential of 10 V during 3 min in a 0.1 M KOH + 10%*v*/*v* H_2_O electrolyte and dissolved in ethylene glycol.

Cu-2p_3/2_			
Chemical Composition	eV	Area	%At Conc
Cu + Cu_2_O	932.2	251,548	42
CuO	933.9	104,503	18
Cu(OH)_2_	936.2	120,911	20
**Cu-** **Auger**			
**Chemical Composition**	**eV**	**Area**	**%At Conc**
Cu + CuO	567.0	48,456	14
Cu_2_O	569.3	103,025	31
**O-1s**			
**Chemical Composition**	**eV**	**Area**	**%At Conc**
OH + Cu(OH)_2_	529.6	17,556	16
Cu_2_O + CuO	531.0	30,615	28
H_2_O	532.5	61,589	56

**Table 2:** Comparison of copper oxide nanoneedles with other metal oxide based photocatalysts previously reported in the scientific literature, their applications and their photocatalytic activity in dye degradation.

Oxides	Copper Oxide Morphology	Copper Oxide Synthesis Type	Composition of the Electrolyte Solution	Application	Light Source	Degradation (%)	Dye	Refs.
Fe_2_O_3_/Cu_2_O	Nanoparticles	Hydrothermal	--	Photocatalysis	Visible light	90	MB	[60]
Cu_2_O/Ag/TiO_2_						93	MB	[61]
Cu_2_O/Ag/TiO_2_		electrodeposition	Cu(NO_3_)_2_ + NaOH + lactic acid			98	MB	[62]
Cu_2_O/Ag/TiO_2_/NFs PAN		Electrospinning	--			99	MB	[63]
Cu_2_O/NFs PAN						60	MB	[63]
P25-TiO_2_ (commercial standard)	--	--			UV light	81	MB	[43]
CuO	Nanoneedles	Solution chemistry in basic media				95	DR and VB	[42]
Cu_2_O/CuO		Chemical-thermal oxidation			Visible light	80	MB	[11]
CuO		Anodization and thermal treatment	KOH	Anticorrosive surface	--	--	--	[39]
CuO		Anodization and thermal treatment	KOH	--				[31]
Cu_2_O		Anodization	KOH					[40]
CuO			KOH	Photocatalisis	Visible light	93	MO	[41]
Cu_2_O/CuO			KOH + 10% H_2_O + NH_4_F + ethylene glycol		Sunlight	88	MB	This study
Cu_2_O/CuO			NaOH + 5% H_2_O + NH_4_F + ethylene glycol			82	MB	This study
Cu_2_O			NaOH + 1% H_2_O + NH_4_F + ethylene glycol			74	MB	This study

## Data Availability

Data available on request.
